# Chronic thromboembolic pulmonary hypertension is an uncommon complication of COVID-19: UK national surveillance and observational screening cohort studies

**DOI:** 10.1183/13993003.01742-2023

**Published:** 2024-08-29

**Authors:** S. Ashwin Reddy, Joseph Newman, Olivia C. Leavy, Hakim Ghani, Joanna Pepke-Zaba, John E. Cannon, Karen K. Sheares, Dolores Taboada, Katherine Bunclark, Allan Lawrie, Cathie L. Sudlow, Colin Berry, James M. Wild, Jane A. Mitchell, Jennifer Quint, Jennifer Rossdale, Laura Price, Luke S. Howard, Martin Wilkins, Naveed Sattar, Philip Chowienczyk, Roger Thompson, Louise V. Wain, Alexander Horsley, Ling-Pei Ho, James D. Chalmers, Michael Marks, Krisnah Poinasamy, Betty Raman, Victoria C. Harris, Linzy Houchen-Wolloff, Christopher E. Brightling, Rachael A. Evans, Mark R. Toshner

**Affiliations:** 1 Royal Papworth Hospital NHS Foundation Trust, Cambridge, UK; 2 University of Cambridge, Cambridge, UK; 3 University of Leicester, Leicester, UK; 4 NIHR Leicester Biomedical Research Centre, The Institute for Lung Health, University of Leicester, Leicester, UK; 5 Sheffield Teaching NHS Foundation Trust and University of Sheffield, Sheffield, UK; 6 University of Edinburgh, Edinburgh, UK; 7 NHS Greater Glasgow and Clyde Health Board, and University of Glasgow, Glasgow, UK; 8 Imperial College London, London, UK; 9 Guy's and St Thomas’ NHS Foundation Trust, London, UK; 10 Royal Brompton Hospital, London, UK; 11 Manchester University NHS Foundation Trust and University of Manchester, Manchester, UK; 12 University of Oxford, Oxford, UK; 13 NHS Tayside and University of Dundee, Dundee, UK; 14 London School of Hygiene and Tropical Medicine, London, UK; 15 Asthma UK and British Lung Foundation Partnership, London, UK; 16 Joint first authors

## Abstract

**Background:**

Pulmonary embolism (PE) is a well-recognised complication of coronavirus disease 2019 (COVID-19) infection, and chronic thromboembolic pulmonary disease with and without pulmonary hypertension (CTEPD/CTEPH) are potential life-limiting consequences. At present the burden of CTEPD/CTEPH is unclear and optimal and cost-effective screening strategies yet to be established.

**Methods:**

We evaluated the CTEPD/CTEPH referral rate to the UK national multidisciplinary team (MDT) during the 2017–2022 period to establish the national incidence of CTEPD/CTEPH potentially attributable to COVID-19-associated PE with historical comparator years. All individual cases of suspected CTEPH were reviewed by the MDT for evidence of associated COVID-19. In a separate multicentre cohort, the risk of developing CTEPH following hospitalisation with COVID-19 was calculated using simple clinical parameters at a median of 5 months post-hospital discharge according to existing risk scores using symptoms, ECG and N-terminal pro-brain natriuretic peptide.

**Results:**

By the second year of the pandemic, CTEPH diagnoses had returned to the pre-pandemic baseline (23.1 *versus* 27.8 cases per month; p=0.252). Of 334 confirmed CTEPD/CTEPH cases, four (1.2%) patients were identified to have CTEPH potentially associated with COVID-19 PE, and a further three (0.9%) CTEPD without PH. Of 1094 patients (mean age 58 years, 60.4% male) hospitalised with COVID-19 screened across the UK, 11 (1.0%) were at high risk of CTEPH at follow-up, none of whom had a diagnosis of CTEPH made at the national MDT.

**Conclusion:**

*A priori* risk of developing CTEPH following COVID-19-related hospitalisation is low. Simple risk scoring is a potentially effective way of screening patients for further investigation.

## Shareable abstract

**Overall rates of CTEPD/CTEPH following hospitalisation with COVID-19 are low, and simplified screening processes using reported breathlessness scores, ECG and NT-proBNP are feasible and may be of significant value** https://bit.ly/4aZEEdK

## Graphical abstract

**Figure:**
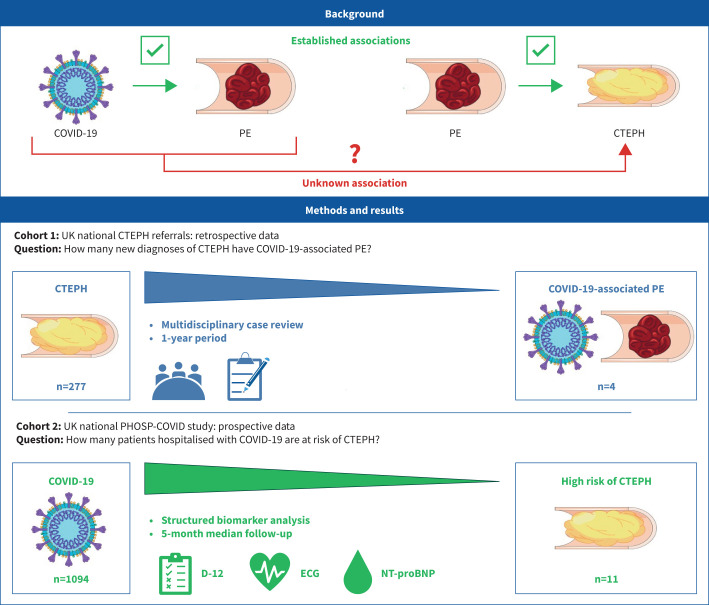
Summary of the background, methods and results of the study, highlighting the two complementary national datasets. COVID-19: coronavirus disease 2019; PE: pulmonary embolism; CTEPH: chronic thromboembolic pulmonary hypertension; PHOSP-COVID: Post-Hospitalisation COVID-19; D-12: Dyspnea-12; NT-proBNP: N-terminal pro-brain natriuretic peptide. Servier Medical Art material used under CC BY 4.0 licence: https://creativecommons.org/licenses/by/4.0

## Introduction

A relationship between coronavirus disease 2019 (COVID-19) and acute pulmonary embolism (PE) has been observed. This is due to endothelial dysfunction and a pro-coagulant inflammatory state [1]. The incidence of PE varies considerably by severity of COVID-19, complicating an average of 3.4% of COVID-19-related hospital admissions overall [2] and up to 26% of COVID-19 patients admitted to intensive care [3].

Chronic thromboembolic pulmonary hypertension (CTEPH) is a rare but potentially life-limiting complication of PE characterised by obstructive remodelling of the pulmonary arteries [4]. Prior to the pandemic, ∼3% of patients who survived a PE later developed CTEPH [5]. The incidence of symptomatic chronic thromboembolic pulmonary disease (CTEPD) where there is significant physiological and symptomatic disease but without resting PH is not currently known. Early identification and diagnosis of CTEPH allows for timelier referral to specialist centres and introduction of treatment, which may improve or cure disease [6]. To facilitate this, others have sought to devise strategies to help rule out CTEPH after acute PE based on non-invasive investigations that comprise standard clinical workup [7, 8].

The relationship between COVID-19 and CTEPH is not yet understood. It seems logical that a proportion of patients will develop chronic disease, but it is unclear if this is to be expected to conform to the same rates as classical causes of PE/CTEPH [9]. Our previous retrospective study reported a decrease in the rate of CTEPH referrals during the first 12 months of the pandemic and identified no cases secondary to COVID-19 [10]. This was potentially due to an overburdened healthcare system and a historical median lag time of 14 months from index PE to CTEPH diagnosis [4]. We have therefore extended this study prospectively to cover the second year of the pandemic. The UK uniquely captures every specialist referral for CTEPD/CTEPH for the whole country because of the nature of the national centrally commissioned service.

The high burden of patients who have developed COVID-19 and the high rates of residual breathlessness [11] have presented challenges in defining effective and efficient ways to investigate patients in overburdened healthcare systems. In a separate multicentre cohort we sought to apply existing risk-scoring strategies to patients who have survived COVID-19-related hospitalisation. The aim of this was to estimate the proportion of COVID-19-hospitalised patients deemed to be at high risk of CTEPH who may require further investigation, in order to inform guidelines and rationalise the use of outpatient diagnostics.

## Methods

Two UK national datasets were interrogated to understand the relationship between COVID-19 and CTEPH.

### COVID-19-associated CTEPH

All cases of suspected CTEPH referred to the national multidisciplinary team (MDT) at the Royal Papworth Hospital (Cambridge, UK) following at least 3 months of effective anticoagulation were contemporaneously reviewed as part of routine standard of care during the second year of the pandemic (2021–2022). The focus was on the second year of the pandemic as no cases of CTEPH associated with COVID-19 had previously been identified for 2020–2021 [10]. All referred patients were assessed by PH physicians, cardiothoracic surgeons, interventional cardiologists and radiologists applying the contemporary CTEPH guidelines [4]. As the Royal Papworth Hospital is the only UK centre that offers pulmonary endarterectomy or balloon pulmonary angioplasty, quaternary-level referrals are received from across the UK and Ireland. Patients with confirmed CTEPD were divided into one of two groups: CTEPH or CTEPD without PH, as per the 2015 European Society of Cardiology/European Respiratory Society guidelines haemodynamic definitions (mean pulmonary arterial pressure ≥25 mmHg, pulmonary arterial wedge pressure ≤15 mmHg, pulmonary vascular resistance >3 WU) [4]. The 2015 guidelines adhere most closely to the established evidence base and clinical commissioning. Monthly national CTEPH referral rates over the 3-year aggregate baseline mean (March 2017 to February 2020) were compared to the first (March 2020 to February 2021) and the second years (March 2021 to February 2022) of the pandemic by one-way ANOVA.

Each CTEPH and CTEPD case was reviewed for evidence of associated COVID-19 based on clinical referral data, serology and thoracic radiology. Association of COVID-19/PE/CTEPH was determined by a clear linear temporal relationship of COVID-19 with concurrent or subsequent PE within 3 months, followed by CTEPH with symptoms following this trajectory in a typical manner and in the absence of other CTEPH risk factors [12]. The likelihood of association was subdivided into “very likely” (concurrent PE or PE within 1 month of COVID-19), “probable” (PE within 3 months of COVID-19) or “unlikely” (PE more than 3 months post-COVID-19). We acknowledge causal association here is challenging and have accordingly been circumspect in ascribing causality. However, the temporality, lack of other risks factors and strong associations of COVID-19 with PE [1] and PE with CTEPH [5] make causality highly likely. The independent adjudication panel, comprised of four clinicians, was required to form a unanimous specialist opinion. UK Health Research Authority ethical approval was not deemed a requirement for this study as it comprised analysis of retrospectively acquired existing anonymised clinical data.

### Non-invasive assessment of CTEPH risk

As part of the PHOSP-COVID (Post-Hospitalisation COVID-19) study [11], demographic and clinical information was prospectively collected on adults discharged from hospital in the UK with a diagnosis of COVID-19 across 83 centres between 1 February 2020 and 31 March 2021 (ethics approval: 20/YH/0225). All patients >18 years of age who attended follow-up assessment a median (range) of 5 (2–7) months post-hospital discharge were eligible for inclusion. Descriptive data for the cohort includes deprivation scores, body mass index (BMI), smoking history, comorbidities, COVID-19 severity indices, COVID-19 treatment and duration of hospitalisation.

At follow-up patients were reviewed sequentially by the following assessments: 1) clinically reported breathlessness, defined as a Dyspnea-12 (D-12) score >0 [13]; 2) ECG evidence of right ventricular pressure overload (rSR′ or rSr′ pattern in lead V1 and/or R:S >1 in lead V1 with R >0.5 mV and/or QRS axis >90°) [14]; and 3) elevated cardiac biomarkers suggestive of ventricular strain, specifically defined as N-terminal pro-brain natriuretic peptide (NT-proBNP) >80 pg·mL^−1^ [8, 15].

Patients were then stratified into one of four groups denoting prospective risk of CTEPH [8]: 1) very low risk (D-12 score 0), 2) low risk (D-12 score >0 but no ECG criteria), 3) intermediate risk (D-12 score >0 and ≥1 ECG criteria but normal NT-proBNP) and 4) high risk (D-12 score >0 and ≥1 ECG criteria and elevated NT-proBNP).

All 12-lead ECGs were scanned into a central study repository and were individually read by trained physicians to determine whether the prespecified right ventricular pressure overload criteria were met. 10% of each physician's reads were cross-checked by a second clinician to ensure accuracy.

### Statistical analysis

Continuous variables are presented as mean with standard deviation or median (interquartile range) and categorical data as count and/or percentage. Comparisons of parametric continuous data were performed using t-tests and ANOVA, whilst categorical data were compared using the Chi-squared test. Comparisons of non-parametric data were performed using the Mann–Whitney U-test or Kruskal–Wallis calculation for multiple-group testing.

Statistical analysis was performed with R version 4.2.3 (www.r-project.org).

## Results

### CTEPH attributable to COVID-19

The pre-pandemic, year 1 pandemic and year 2 pandemic populations were similar in terms of age and sex (table 1). There was no difference between the mean CTEPH referral rate during the second year of the pandemic compared to the pre-pandemic baseline (23.1 *versus* 27.8 cases per month; p=0.252) (table 1).

**TABLE 1:** Chronic thromboembolic pulmonary hypertension (CTEPH) referral numbers, provenance and demographics for pre-pandemic and pandemic years

	Pre-pandemic				Pandemic			
	Year 1 (Mar 2017 –Feb 2018)	Year 2 (Mar 2018 –Feb 2019)	Year 3 (Mar 2019 –Feb 2020)	3-year aggregate baseline (Mar 2017 –Feb 2020)	Year 1 (Mar 2020 –Feb 2021)	p-value *versus* baseline	Year 2 (Mar 2021 –Feb 2022)	p-value *versus* baseline
**Total CTEPH diagnoses, n**	328	327	345	1000	228		277	
**Mean CTEPH diagnoses per month, n**	27.3	27.3	28.8	27.8	19	0.010	23.1	0.252
**Mean age of patient, years**	60.4	61.4	60.9	60.9	60.6	0.975	59.6	0.437
**Male, %**	50.9	50.5	52.8	51.4	52.2	0.829	48.70	0.433
**Direct PEA clinic review, %**	49.7	47.1	51.9	49.6	60.1	0.004	47.30	0.497
**Total associated COVID-19 cases, n**					0		4	
**Referrals from each PH centre, % (n)**								
Belfast and Dublin	3.7 (12)	2.8 (9)	3.2 (11)	3.2 (32)	4.4 (10)		2.5 (7)	
Golden Jubilee National Hospital, Glasgow	8.5 (28)	8.4 (31)	4.9 (17)	7.6 (76)	9.2 (21)		9.4 (26)	
Hammersmith Hospital, London	7.6 (25)	8.9 (33)	12.2 (42)	10 (100)	8.8 (20)		9.4 (26)	
Freeman Hospital, Newcastle	7.0 (23)	6.1 (20)	3.8 (13)	5.6 (56)	7.0 (16)		4.3 (12)	
Royal Brompton Hospital, London	3.4 (11)	5.8 (19)	6.4 (22)	5.3 (52)	6.6 (15)		9.7 (27)	
Royal Papworth Hospital, Cambridge	30.5 (100)	27.8 (91)	26.1 (90)	28.1 (281)	25 (57)		28.5 (79)	
Royal Free Hospital, London	12.8 (42)	14.1 (46)	16.5 (57)	14.5 (145)	10.5 (24)		10.5 (29)	
Royal Hallamshire Hospital, Sheffield	26.5 (87)	23.9 (78)	27.0 (93)	25.8 (258)	28.5 (65)		25.3 (71)	

PEA: pulmonary endarterectomy; COVID-19: coronavirus disease 2019; PH: pulmonary hypertension.

**FIGURE 1:**
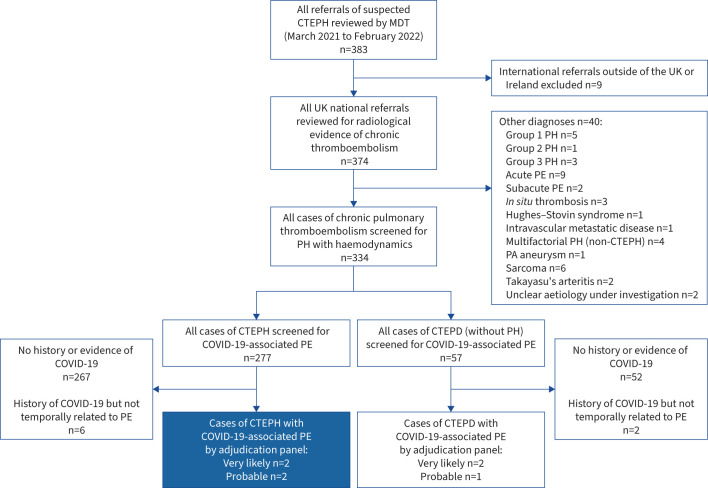
Flowchart showing the decision-making process to associate coronavirus disease 2019 (COVID-19) infection with subsequent chronic thromboembolic pulmonary hypertension (CTEPH). MDT: multidisciplinary team; PH: pulmonary hypertension; PE: pulmonary embolism.

**FIGURE 2:**
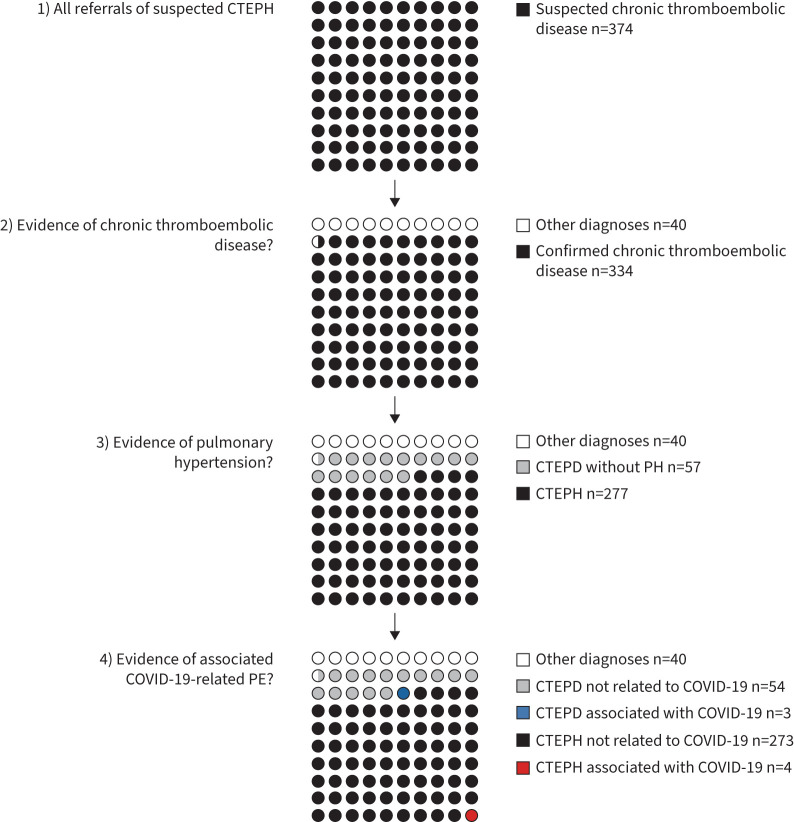
Illustration of the proportion of chronic thromboembolic pulmonary disease (CTEPD) referrals potentially related to coronavirus disease 2019 (COVID-19). CTEPH: chronic thromboembolic pulmonary hypertension; PE: pulmonary embolism.

383 cases of suspected CTEPH were referred during the second year of the pandemic (figure 1). Nine international cases were excluded and 40 had an alternative diagnosis. Of the remaining 334 cases, 277 had CTEPH (figure 2). Six had a history of COVID-19 not temporally related to their diagnosis of CTEPH. Four patients (1.4% of all confirmed CTEPH cases) were identified to have CTEPH with COVID-19-associated PE without other obvious contributing risk factors. In each case the PE caused typical symptoms, was diagnosed by computed tomography (CT) pulmonary angiogram and treated with ≥3 months of anticoagulation. The adjudicating panel judged the overall likelihood of causation in these cases to be “very likely” in two cases and “probable” in two cases. The diagnosis of first PE was <1 and <3 months following COVID-19, respectively [9]. There were also three cases (two “very likely” and one “probable”) of CTEPD without PH with COVID-19-associated PE [8].

Of these four novel CTEPH cases associated with COVID-19 PE, three were female with a mean age of 52.3 years (table 2). Three of the patients had mild index COVID-19 and one case was moderate, as per the World Health Organization classification [16]. The time from index COVID-19 episode to CTEPH diagnosis ranged from 5 to 21 months. The radiological distribution of disease was proximal in all four cases, and all four patients have undergone pulmonary endarterectomy surgery. None of these cases were from the prospective observational study (PHOSP-COVID), and in our previous work covering the first year of the pandemic no COVID-associated CTEPH cases were diagnosed [9].

**TABLE 2:** Baseline characteristics of newly diagnosed chronic thromboembolic pulmonary hypertension (CTEPH) and chronic thromboembolic pulmonary disease (CTEPD) patients over the study period (March 2021 to February 2022) with and without coronavirus disease 2019 (COVID-19)-associated pulmonary embolism

	CTEPH			CTEPD without PH		
	All	Not associated with COVID-19	Associated with COVID-19	All	Not associated with COVID-19	Associated with COVID-19
**Total**	277 (83)	273	4	57 (17.1)	54	3
**Age, years**	59.6±14.8	59.7±14.7	52.3	56.1±17.4	57.2±16.8	35.3
**Sex at birth**						
Male	135 (49)	134	1	28 (49)	27	1
Female	142 (51)	139	3	29 (51)	27	2
**BMI, kg·m^−2^**	29.1 (24.9–35.9)	29.0 (24.9–35.4)	37.1	30.1 (26.0–35.0)	30.0 (26.0–34.2)	44.3
BMI <30 kg·m^−2^	135 (52.3)	134	1	28 (49.1)	27 (50)	1
BMI ≥30 kg·m^−2^	123 (47.7)	120	3	29 (50.9)	27 (50)	2
Missing	19	19	0	0	0	0
**Comorbidities, n**	1 (0–2)	1 (0–2)	0.5	1 (1–2)	1 (1–2)	1
No comorbidity	76 (28.0)	74 (27.3)	2	14 (24.6)	13 (24.1)	1
1 comorbidity	99 (36.5)	97 (35.8)	2	25 (43.9)	23 (42.6)	2
≥2 comorbidities	96 (35.4)	96 (35.4)	0	18 (31.6)	18 (33.3)	0
Missing	6	6	0	0	0	0
**Baseline haemodynamics**						
mPAP, mmHg	42.5±11.5	42.5±11.6	42.5	21.4±7.7	21.4±7.7	21.3
PVR, WU	7.7±4.6	8.0±5.6	6	2.0±1.2	2.0±1.2	2.2
PCWP, mmHg	9.9±4.2	9.9±4.2	9.5	9.5±3.2	9.6±3.1	8.0
Cardiac output, L·min^−1^	4.6±1.5	4.6±1.5	5.9	5.7±1.3	5.7±1.4	6.0
**Baseline 6MWD, m**	301±153	303±154	215	352±117	349±118	392
**Baseline CAMPHOR score**						
Symptoms	12±7	12±7	16	12±7	12±7	
Activity	11±7	11±7	7	9±7	10±7	
Quality of life	11±7	10±7	16	9±7	9±7	
Total	34±19	33±20	30	29±19	30±19	
Missing	161	160	1	27	25	2
**Baseline NYHA FC**						
I	4 (1.6)	4 (1.6)	0	1 (1.8)	1 (1.9)	0
II	43 (17.2)	43 (17.5)	0	20 (35.1)	18 (33.3)	2
III	191 (76.4)	187 (76.0)	4	34 (61.4)	33 (61.1)	1
IV	12 (4.8)	12 (4.9)	0	1 (1.8)	1 (1.9)	0
Missing	27	27	0	1	1	0
**Initial MDT management decision**						
For PEA clinic review	131 (47.3)	127 (46.5)	4	7 (12.3)	7 (13.0)	0
For BPA clinic review	30 (10.8)	30 (11.0)	0	1 (1.8)	1 (1.9)	0
Not for intervention	116 (41.9)	116 (42.5)	0	49 (86.0)	46 (85.2)	3

Data are presented as n (%), mean±sd, median (interquartile range) or n. BMI: body mass index; mPAP: mean pulmonary arterial pressure; PVR: pulmonary vascular resistance; PCWP: pulmonary capillary wedge pressure; 6MWD: 6-min walk distance; CAMPHOR: Cambridge Pulmonary Hypertension Outcome Review; NYHA FC: New York Heart Association Functional Class; MDT: multidisciplinary team; PEA: pulmonary endarterectomy; BPA: balloon pulmonary angioplasty.

### CTEPH risk evaluation following COVID-19 hospitalisation

Over the study period, 1094 patients across the study sites attended secondary care follow-up a median (range) of 5 (2–7) months post-hospital discharge with COVID-19 as part of the PHOSP-COVID study, and had ECG and clinical data available for review by the study team. The average age of this group was 58 years and 659 (60.4%) were male. The mean±sd duration of hospitalisation was 12.6±16.8 days. 66% of the cohort required supplemental oxygen during their admission, whilst a further 15% had required intensive care support.

**FIGURE 3:**
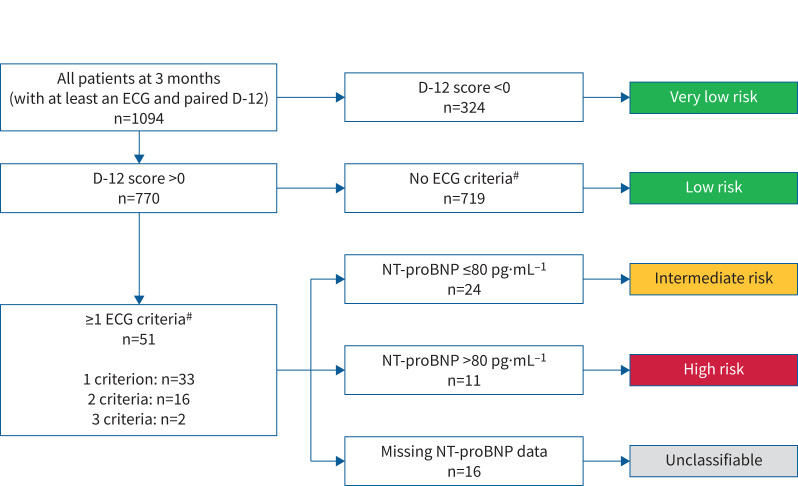
Flowchart showing the sequential assessment of patients at 3 months post-hospitalisation with coronavirus disease 2019 by Dyspnea-12 (D-12) score, ECG and N-terminal pro-brain natriuretic peptide (NT-proBNP) to risk stratify into very-low-, low-, intermediate- and high-risk categories. ^#^: ECG criteria: rSR′ or rSr′ pattern in lead V1; and/or R:S >1 in lead V1 with R >0.5 mV; and/or QRS axis >90°.

On review of clinical variables at 3-month follow-up, 324 patients (29.6%) reported no breathlessness and thus were deemed very low risk of developing CTEPH according to the InShape II criteria [8]. 719 (65.7%) reported breathlessness (D-12 score >0), but had no ECG features of right ventricular strain, so were deemed low risk. Among the 51 patients who reported breathlessness and demonstrated at least one feature of right ventricular strain on their ECG, 24 (2.2%) had a normal NT-proBNP, so were deemed intermediate risk, whilst 11 (1.0%) had elevated NT-proBNP and thus were classified as high risk of developing PH. The remaining 16 patients did not have NT-proBNP assay results available to view and were accordingly deemed unclassifiable, although they were of at least intermediate risk (figure 3).

The demographics of the entire study population, and classified according to risk status, are shown in table 3. There was no significant difference in age or sex between low- and high-risk groups. Patients classified as high risk for developing CTEPH had significantly longer mean±sd inpatient hospital admission (40±45 days for high risk *versus* 12±16, 13±16 and 15±16 days for very low, low and intermediate risk, respectively; p<0.001).

**TABLE 3:** Data table of all participants in the study and by risk stratification (very low, low, intermediate or high risk) including demographics, comorbidities and descriptive data of the index coronavirus disease 2019 admission

Variable	Total	High risk	Intermediate risk	Low risk	Very low risk	Total
**Total**		11 (1.0)	24 (2.2)	719 (66.7)	324 (30.1)	1078
**Age, years**	1078 (100.0)	62.6±17.1	60.0±11.4	57.7±12.1	59.8±14.0	58.4±12.8
**Sex at birth**	1077 (99.9)					
Male		5 (45.5)	17 (70.8)	398 (55.4)	228 (70.4)	648 (60.2)
Female		6 (54.5)	7 (29.2)	320 (44.6)	96 (29.6)	429 (39.8)
Missing		0	0	<5	0	<5
**Ethnicity**	1073 (99.5)					
White		10 (90.9)	22 (91.7)	576 (80.7)	245 (75.6)	853 (79.5)
South Asian		0 (0.0)	<5	75 (10.5)	43 (13.3)	119 (11.1)
Afro-Caribbean		<5	0 (0.0)	36 (5.0)	22 (6.8)	59 (5.5)
Mixed		0 (0.0)	0 (0.0)	11 (1.5)	5 (1.5)	16 (1.5)
Other		0 (0.0)	<5	16 (2.2)	9 (2.8)	26 (2.4)
Missing		0	0	5	0	5
**Index of Multiple Deprivation**	1076 (99.8)					
1 (most deprived)		0 (0.0)	6 (25.0)	143 (19.9)	64 (19.8)	213 (19.8)
2		<5	<5	167 (23.3)	58 (18.0)	231 (21.5)
3		<5	<5	122 (17.0)	46 (14.2)	171 (15.9)
4		<5	7 (29.2)	126 (17.5)	73 (22.6)	207 (19.2)
5 (least deprived)		<5	8 (33.3)	160 (22.3)	82 (25.4)	254 (23.6)
Missing		0	0	<5	<5	<5
**BMI, kg·m^−2^**	957 (88.8)	30.4 (24.1–35.7)	32.0 (29.6–35.5)	31.7 (28.2–36.7)	30.0 (26.5–33.8)	31.2 (27.7–35.9)
BMI <30 kg·m^−2^		5 (45.5)	7 (30.4)	245 (37.9)	138 (49.8)	395 (41.3)
BMI ≥30 kg·m^−2^		6 (54.5)	16 (69.6)	401 (62.1)	139 (50.2)	562 (58.7)
Missing		0	<5	73	47	121
**Cigarette smoking**	1010 (93.7)					
Never-smoker		<5	7 (30.4)	354 (52.3)	176 (58.7)	541 (53.6)
Ex-smoker		6 (60.0)	16 (69.6)	308 (45.5)	119 (39.7)	449 (44.5)
Current smoker		0 (0.0)	0 (0.0)	15 (2.2)	5 (1.7)	20 (2.0)
Missing		<5	<5	42	24	68
**WHO Clinical Progression Scale**	1078 (100.0)					
3–4		0 (0.0)	<5	134 (18.6)	65 (20.1)	203 (18.8)
5		<5	5 (20.8)	289 (40.2)	148 (45.7)	446 (41.4)
6		<5	10 (41.7)	184 (25.6)	69 (21.3)	266 (24.7)
7–9		<5	5 (20.8)	112 (15.6)	42 (13.0)	163 (15.1)
**Number of comorbidities**	1078 (100.0)	3 (1.5–3.5)	3 (1–4)	2 (1–4)	1 (0–1)	2 (1–3)
No comorbidity		0 (0.0)	<5	148 (20.6)	90 (27.8)	241 (22.4)
1 comorbidity		<5	<5	141 (19.6)	75 (23.1)	223 (20.7)
≥2 comorbidities		8 (72.7)	17 (70.8)	430 (59.8)	159 (49.1)	614 (57.0)
**Admission duration, days**	1078 (100.0)	40.1±45.0	15.0±15.1	12.6±16.1	11.8±16.1	12.7±16.8
**SARS-CoV-2 swab**	1008 (93.5)					
Negative		0 (0.0)	<5	47 (7.0)	18 (5.9)	66 (6.5)
Positive		11 (100.0)	21 (95.5)	620 (92.7)	285 (93.1)	937 (93.0)
Indeterminate		0 (0.0)	0 (0.0)	<5	<5	5 (0.5)
Missing		0	<5	50	18	70
**Systemic (oral or *i.v.*) steroids**	1040 (96.5)					
No		<5	8 (34.8)	316 (45.5)	159 (51.1)	486 (46.7)
Yes		8 (72.7)	15 (65.2)	379 (54.5)	152 (48.9)	554 (53.3)
Missing		0	<5	24	13	38

Data are presented as n (%), mean±sd, n or median (interquartile range). BMI: body mass index; WHO: World Health Organization; SARS-CoV-2: severe acute respiratory syndrome coronavirus 2; *i.v.*: intravenous. The difference in totals between the overall cohort (n=1094) and the sum of the four risk-stratified groups (n=1078) is due to patients being excluded as “unclassifiable” risk scores due to missing N-terminal pro-brain natriuretic peptide data, as shown in figure 3. Data pertaining to less than five individuals are shown as “<5” to ensure patients remain non-identifiable.

## Discussion

We observe that only a very small proportion of new CTEPH diagnoses are likely to be attributable to COVID-19-associated PE, and also that very few patients hospitalised with COVID-19 are high risk for developing CTEPH at follow-up. This is despite national UK CTEPH referrals returning to pre-pandemic baseline rates. In a separate multicentre cohort we demonstrate that a simple screening algorithm can be applied to determine patients who may need further evaluation.

Our data agree with de Jong *et al*. [17], who screened 299 patients who had a diagnosis of COVID-19-associated PE in 13 Dutch hospitals without finding any cases of CTEPH. Our work therefore clarifies that in addition to patients with COVID-19-associated PE having low rates of CTEPH, low rates of referrals have been seen across the whole UK healthcare system, and by applying widely available, cheap clinical tests to patients post-COVID, we can simplify assessment and risk stratification.

As time has elapsed since the onset of the COVID-19 pandemic the knowledge base of long-term sequelae associated with the condition continues to grow. Concurrently, new viral strains continue to appear as COVID-19 evolves and the impact of this is not yet clear. PE has been shown to complicate ∼0.5% of COVID-19 sufferers, an incidence nearly ninefold higher than in those who have not contracted COVID-19 [18]. Endothelial dysfunction associated with COVID-19 disease gives rise to local inflammation and hypercoagulability, and thrombosis formation that is thought to be predominantly *in situ* rather than thromboembolic [19, 20]. An early case series evaluating necropsy specimens revealed significantly more widespread histological microangiopathy and intussusceptive angiogenesis along with extensive capillary microthrombi in seven patients who died following COVID-19 infection compared to seven necropsy specimens obtained from patients who died of influenza [21]. It not known for certain, though, whether subsequent viral strains mediate the same pathological effect. PE associated with COVID-19 is significantly less likely to be found in the main pulmonary artery and is more commonly seen in segmental branches when compared to non-COVID-19 patients [22]. The microscopic, distal nature of pulmonary vascular obstruction in COVID-19 can be difficult to identify with CT or scintigraphy [23]. It is thus possible that CTEPH may occur in COVID-19 patients without detectable PE, and it is therefore important to have a low index of suspicion to screen for this in symptomatic individuals. Further investigation for the presence of chronic thrombus should be undertaken even when anticoagulated, since delays to CTEPH diagnosis correlate with significantly higher morbidity and mortality, which may be abrogated by prompt initiation of PH-specific treatment [4]. Our findings suggest that only a small proportion of patients will require screening for CTEPH with more advanced imaging and invasive testing based on existing easily implementable risk calculation [8].

The low observed prospective risk of CTEPH in hospitalised COVID-19 patients is manifest by the absence of a spike of CTEPH referrals to the national intervention centre during the study period despite the very high incidence of COVID-19 nationally, a crude indicator that there has not been a major influx of CTEPH due to COVID-19 disease. This is not explained by a lower overall rate of referral due to an overburdened healthcare system as we note that the national CTEPH referral rate has rebounded to pre-pandemic levels, reflecting a recovery of referral pathways. The specific incidence of CTEPH after COVID-19 has been sparsely reported in the wider literature. In a small study by Cueto-Robledo *et al*. [24], three cases of CTEPH were attributed to COVID-19-associated PE out of 77 total PE diagnoses over an 11-month period. The incidence of 3.8% is comparable to the rate observed in non-COVID-19-associated PE [25, 26]. This contrasts with de Jong *et al*. [17], whose findings of no CTEPH cases among 299 patients with COVID-19-associated PE derived from multiple Dutch centres more closely mirror our own observed incidence of 1.4%. It is probable that although rare, this emerging disease entity is underdiagnosed given the high incidence of COVID-19-associated PE over the past 2 years. There are many potential explanations for this. Primarily, CTEPH is a rare disease and thus we acknowledge that the issue may just be variance rather than a genuine difference in point estimates of incidence. Mortality bias may be a key contributor as we found COVID-19 patients who were at highest risk of developing CTEPH at 3 months were those with the most severe disease at baseline, requiring longer hospital stay and more advanced treatment, and thus many may not have survived to develop CTEPH. In addition, patients with ongoing symptoms after COVID-19 recovery may have been given alternative diagnoses, including “long COVID”, and as yet there are no structured guidelines for excluding CTEPH. As mentioned, a high index of suspicion is required to investigate for latent CTEPH to avoid omitting or delaying disease-specific treatment, hence the potential value of screening. We await evidence from COVID-19-associated PE cohorts to better define the incidence of, and risk factors for, CTEPH. This may further contribute to our understanding of why we have seen so few cases progress to CTEPH.

None of the current work has yet ascertained CTEPD rates. Research regarding thromboprophylaxis in COVID-19 is also clearly of importance.

### Limitations

This national CTEPH dataset is a retrospective analysis, though one that captures a relatively complete dataset from a whole country. The adjudication of whether or not PE, and subsequent CTEPH, was related to COVID-19 in our study was necessarily subjective as no objective criteria are yet established. We also acknowledge that over the subsequent time period more widespread exposure to COVID-19, due to relaxation of quarantine rules, introduction of antiviral therapy and deployment of vaccines, resulted in the virus becoming more “endemic”, making it potentially challenging to establish whether PE was related to COVID-19 infection or spontaneous. We have mitigated this by enforcing strict criteria for the diagnosis of COVID-19-related PE and stipulating that all four clinicians on the independent adjudication panel required unanimous agreement on the diagnosis and limiting the follow-up period to the first 2 years of the pandemic, thus reducing the risk of ascertainment bias. We also note that no change in overall diagnostic rates has been observed, which agrees with the low rates of COVID-19-associated CTEPH seen. Some cases of CTEPH may not have been referred to the national MDT. Finally, the InShape II criteria were not specifically designed for the purpose of risk stratifying the post-COVID-19 patient population.

### Conclusions

CTEPD/CTEPH following hospitalisation with COVID-19 remains a differential diagnosis that should be considered in the chronically breathless patient. Our work, however, adds to the literature that suggests that overall rates are not high, and that simplified screening processes using reported breathlessness scores, ECG and NT-proBNP are feasible and may be of significant value.

## Supplementary material

10.1183/13993003.01742-2023.Supp1 **Please note:** supplementary material is not edited by the Editorial Office, and is uploaded as it has been supplied by the author.
Supplementary file: list of members of the PHOSP-COVID Collaborative Group ERJ-01742-2023.Supplement

## Shareable PDF

10.1183/13993003.01742-2023.Shareable1 This one-page PDF can be shared freely online.
Shareable PDF ERJ-01742-2023.Shareable
