# Supplementary material for: Chronic thromboembolic pulmonary hypertension is an uncommon complication of COVID-19: UK national surveillance and observational screening cohort studies
Source: Eur Respir J. 2024 Aug 29;64(2):2301742. doi: 10.1183/13993003.01742-2023 (PMC11358513; doi:10.1183/13993003.01742-2023)
Supplement: Supplementary file 2 [file ERJ-01742-2023.Shareable.pdf]

EUROPEAN RESPIRATORY JOURNAL
ORIGINAL RESEARCH ARTICLE
S.A. REDDY ET AL.

SHAREABLE PDF

Chronic thromboembolic pulmonary hypertension is an uncommon complication of COVID-19: UK national surveillance and observational screening cohort studies

S. Ashwin Reddy, Joseph Newman, Olivia C. Leavy, *et al.*

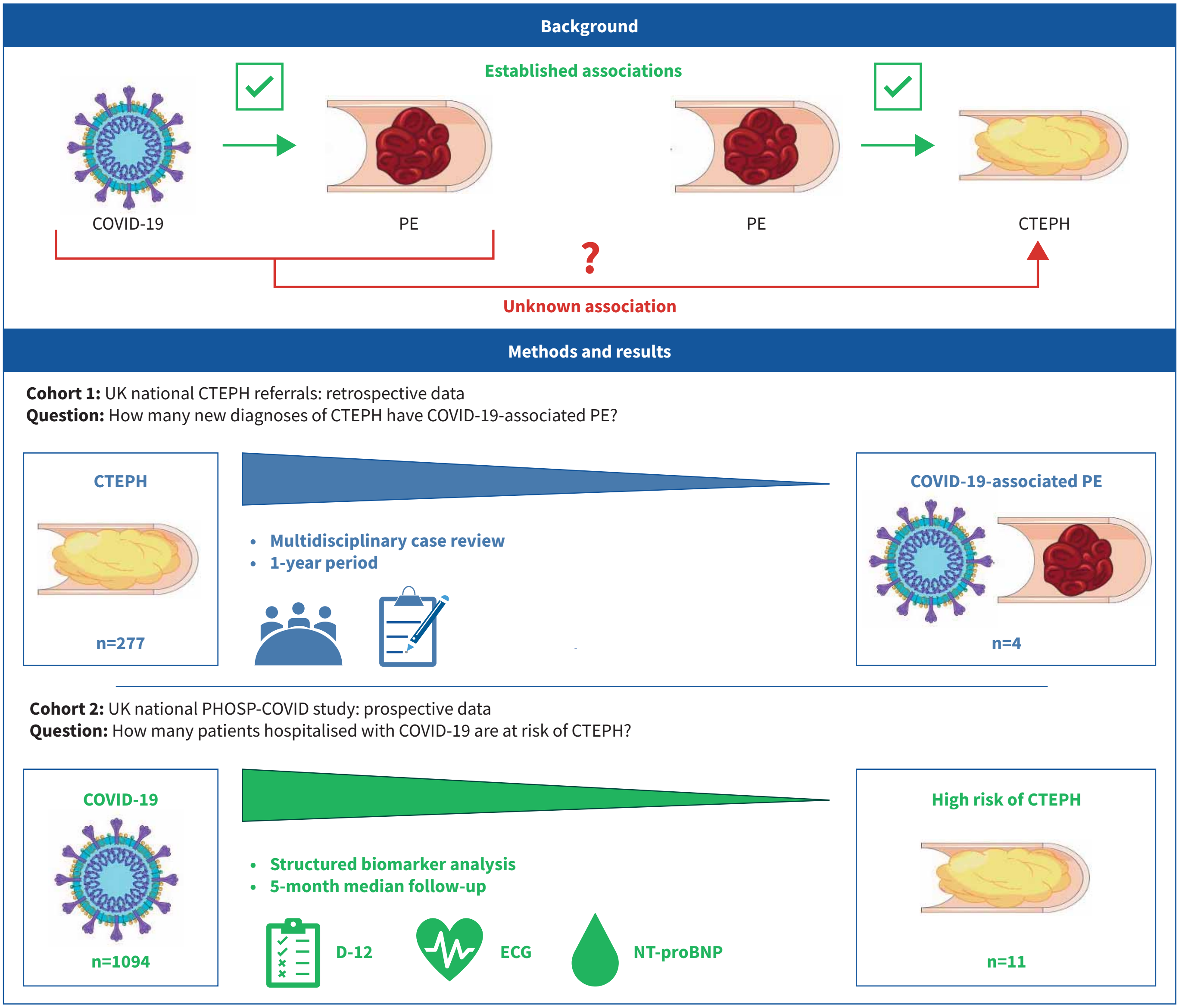

GRAPHICAL ABSTRACT Summary of the background, methods and results of the study, highlighting the two complementary national datasets. COVID-19: coronavirus disease 2019; PE: pulmonary embolism; CTEPH: chronic thromboembolic pulmonary hypertension; PHOSP-COVID: Post-Hospitalisation COVID-19; D-12: Dyspnea-12; NT-proBNP: N-terminal pro-brain natriuretic peptide. Servier Medical Art material used under CC BY 4.0 licence: https://creativecommons.org/licenses/by/4.0

Link to published version: https://doi.org/10.1183/13993003.01742-2023

EUROPEAN RESPIRATORY JOURNAL
ORIGINAL RESEARCH ARTICLE
S.A. REDDY ET AL.

SHAREABLE PDF

Chronic thromboembolic pulmonary hypertension is an uncommon complication of COVID-19: UK national surveillance and observational screening cohort studies

S. Ashwin Reddy [1,2,16], Joseph Newman [1,2,16], Olivia C. Leavy[3,4], Hakim Ghani[1,2], Joanna Pepke-Zaba[1], John E. Cannon[1], Karen K. Sheares[1], Dolores Taboada[1], Katherine Bunclark[1], Allan Lawrie [5], Cathie L. Sudlow[6], Colin Berry[7], James M. Wild[5], Jane A. Mitchell[8], Jennifer Quint [8], Jennifer Rossdale[9], Laura Price [8,10], Luke S. Howard [8], Martin Wilkins [8], Naveed Sattar[7], Philip Chowienczyk[10], Roger Thompson [5], Louise V. Wain [4], Alexander Horsley [11], Ling-Pei Ho[12], James D. Chalmers[13], Michael Marks [14], Krisnah Poinasamy[15], Betty Raman [12], Victoria C. Harris[3], Linzy Houchen-Wolloff[3], Christopher E. Brightling[4], Rachael A. Evans [4] and Mark R. Toshner [1,2] on behalf of the PHOSP-COVID Study Collaborative Group

[1]Royal Papworth Hospital NHS Foundation Trust, Cambridge, UK. [2]University of Cambridge, Cambridge, UK. [3]University of Leicester, Leicester, UK. [4]NIHR Leicester Biomedical Research Centre, The Institute for Lung Health, University of Leicester, Leicester, UK. [5]Sheffield Teaching NHS Foundation Trust and University of Sheffield, Sheffield, UK. [6]University of Edinburgh, Edinburgh, UK. [7]NHS Greater Glasgow and Clyde Health Board, and University of Glasgow, Glasgow, UK. [8]Imperial College London, London, UK. [9]Guy's and St Thomas' NHS Foundation Trust, London, UK. [10]Royal Brompton Hospital, London, UK. [11]Manchester University NHS Foundation Trust and University of Manchester, Manchester, UK. [12]University of Oxford, Oxford, UK. [13]NHS Tayside and University of Dundee, Dundee, UK. [14]London School of Hygiene and Tropical Medicine, London, UK. [15]Asthma UK and British Lung Foundation Partnership, London, UK. [16]Joint first authors.

Corresponding author: Mark R. Toshner (mrt34@medschl.cam.ac.uk)

Check for updates

Shareable abstract (@ERSpublications)
Overall rates of CTEPD/CTEPH following hospitalisation with COVID-19 are low, and simplified screening processes using reported breathlessness scores, ECG and NT-proBNP are feasible and may be of significant value https://bit.ly/4aZEEdK

Cite this article as: Reddy SA, Newman J, Leavy OC, *et al*. Chronic thromboembolic pulmonary hypertension is an uncommon complication of COVID-19: UK national surveillance and observational screening cohort studies. *Eur Respir J* 2024; 64: 2301742 [DOI: 10.1183/13993003.01742-2023].

This extracted version can be shared freely online.

Copyright ©The authors 2024.

This version is distributed under the terms of the Creative Commons Attribution Licence 4.0.

This article has an editorial commentary: https://doi.org/10.1183/13993003.01467-2024

Received: 12 Oct 2023
Accepted: 9 June 2024

Abstract

Background Pulmonary embolism (PE) is a well-recognised complication of coronavirus disease 2019 (COVID-19) infection, and chronic thromboembolic pulmonary disease with and without pulmonary hypertension (CTEPD/CTEPH) are potential life-limiting consequences. At present the burden of CTEPD/CTEPH is unclear and optimal and cost-effective screening strategies yet to be established.

Methods We evaluated the CTEPD/CTEPH referral rate to the UK national multidisciplinary team (MDT) during the 2017–2022 period to establish the national incidence of CTEPD/CTEPH potentially attributable to COVID-19-associated PE with historical comparator years. All individual cases of suspected CTEPH were reviewed by the MDT for evidence of associated COVID-19. In a separate multicentre cohort, the risk of developing CTEPH following hospitalisation with COVID-19 was calculated using simple clinical parameters at a median of 5 months post-hospital discharge according to existing risk scores using symptoms, ECG and N-terminal pro-brain natriuretic peptide.

Results By the second year of the pandemic, CTEPH diagnoses had returned to the pre-pandemic baseline (23.1 *versus* 27.8 cases per month; p=0.252). Of 334 confirmed CTEPD/CTEPH cases, four (1.2%) patients were identified to have CTEPH potentially associated with COVID-19 PE, and a further three (0.9%) CTEPD without PH. Of 1094 patients (mean age 58 years, 60.4% male) hospitalised with COVID-19 screened across the UK, 11 (1.0%) were at high risk of CTEPH at follow-up, none of whom had a diagnosis of CTEPH made at the national MDT.

Conclusion *A priori* risk of developing CTEPH following COVID-19-related hospitalisation is low. Simple risk scoring is a potentially effective way of screening patients for further investigation.
